# Integrated clinical and quality improvement coaching in Son La Province, Vietnam: a model of building public sector capacity for sustainable HIV care delivery

**DOI:** 10.1186/s12913-015-0935-8

**Published:** 2015-07-17

**Authors:** Lisa A. Cosimi, Huong V. Dam, Thai Q. Nguyen, Huyen T. Ho, Phuong T. Do, Duat N. Duc, Huong T. Nguyen, Bridget Gardner, Howard Libman, Todd Pollack, Lisa R. Hirschhorn

**Affiliations:** Brigham and Women’s Hospital, 75 Francis St, Boston, MA 02115 USA; The Partnership for Health Advancement in Vietnam, Beth Israel Deaconess Medical Center, 1309 Beacon St, Brookline, MA USA; Son La Department of Health, Son La Province, Vietnam; The Partnership for Health Advancement in Vietnam, Beth Israel Deaconess Medical Center, 57 Ly Nam De St, Hanoi, Vietnam; Ariadne Labs, a joint Partnership with Brigham and Women’s Hospital and Harvard School of Public Health, Landmark Center, Boston, MA USA; Harvard Medical School, Boston, MA USA

**Keywords:** Quality improvement, Coaching, Teamwork, Resource limited

## Abstract

**Background:**

The global scale-up of antiretroviral therapy included extensive training and onsite support to build the capacity of HIV health care workers. However, traditional efforts aimed at strengthening knowledge and skills often are not successful at improving gaps in the key health systems required for sustaining high quality care.

**Methods:**

We trained and mentored existing staff of the Son La provincial health department and provincial HIV clinic to work as a provincial coaching team (PCT) to provide integrated coaching in clinical HIV skills and quality improvement (QI) to the HIV clinics in the province. Nine core indicators were measured through chart extraction by clinic and provincial staff at baseline and at 6 month intervals thereafter. Coaching from the team to each of the clinics, in both QI and clinical skills, was guided by results of performance measurements, gap analyses, and resulting QI plans.

**Results:**

After 18 months, the PCT had successfully spread QI activities, and was independently providing regular coaching to the provincial general hospital clinic and six of the eight district clinics in the province. The frequency and type of coaching was determined by performance measurement results. Clinics completed a mean of five QI projects. Quality of HIV care was improved throughout all clinics with significant increases in seven of the indicators. Overall both the PCT activities and clinic performance were sustained after integration of the model into the Vietnam National QI Program.

**Conclusions:**

We successfully built capacity of a team of public sector health care workers to provide integrated coaching in both clinical skills and QI across a province. The PCT is a feasible and effective model to spread and sustain quality activities and improve HIV care services in a decentralized rural setting.

## Background

Scale-up of HIV care and treatment has resulted in an estimated 9.7 million people on treatment in lower- and middle-income countries [1]. Training and on-site clinical mentoring have been important components of many national scale-up efforts [2–6]. However, it is increasingly recognized that strengthening healthcare systems in addition to clinical knowledge is necessary to ensure quality of care delivery, long-term sustainability and universal access to effective treatment [7, 8].

Addressing health system gaps at the clinic and local level are beyond the scope of traditional HIV training, clinical coaching, and other continuing medical education approaches that focus on health care worker (HCW) knowledge and skills. Published reports on training and clinical coaching have generally shown limited impact on quality of care, with modest, short-term improvements seen in single performance measures such as prescribing practices, tuberculosis and sexually transmitted infection screening [9–14].

Increasingly, work to improve quality of HIV care in resource-limited settings has aimed to build capacity for systems-focused improvement [4, 7, 15, 16]. However, implementation of many quality improvement (QI) programs has been hindered by factors including human resource shortages and turnover, lack of knowledge and leadership in QI, lack of material resources, and competing health care delivery priorities [17, 18]. While there have been examples of effective scale-up of QI-focused programs in public sectors, published reports of broader sustained improvements in quality of care in low- and middle-income settings remain limited [16, 19–21]. Additional models that systematically strengthen the quality of healthcare delivery are needed.

Vietnam is a lower middle-income country with a population of approximately 90 million and an estimated HIV prevalence of 0.4 % [22]. By the end of 2013, 80,000 persons were receiving treatment through a network of hospital and community-based clinics located throughout the country’s 63 provinces [23]. As in many countries, the initial delivery and scale-up of treatment services was supported by intensive efforts, including didactic training and onsite coaching, to build the clinical skills of HCWs working in the HIV clinics. However, this initial support was not designed to increase HCW and facility capacity to diagnose and improve systems-level gaps that threaten quality. We describe the initial results of a collaboration with provincial health officials in the remote province of Son La, Vietnam designed to build capacity of a team of provincial HCWs and public health staff to provide coaching in clinical skills and in QI. The purpose of the study was to understand whether a provincial coaching team (PCT) model built within the existing infrastructure of the public healthcare system could improve the quality of HIV care throughout the province.

## Methods

### Setting

Son La is a large mountainous province located in North Vietnam along the Lao border. It has a population of 1,092,700 and a per capita income approximately half the national average [24]. The province has one of the highest HIV prevalence rates in the country (0.6 %) [23]. HIV care is delivered through nine HIV outpatient clinics. The largest clinic, opened in 2009, is located at Son La Provincial Hospital (Provincial clinic), and in 2013 provided care to 700 patients. Training of HIV providers in this clinic started with an in-service didactic training in 2009 focused on the care and treatment of HIV-infected patients, followed by quarterly clinical mentoring by HIV clinical experts. The eight remaining clinics are located in smaller district-level health care facilities, the furthest of which is a 4-h drive from the provincial capital. Staff working in these clinics also attended an in-service didactic HIV training and received periodic clinical skills coaching from the Provincial clinic physicians. Son La government health staff from the Provincial People’s AIDS Committee (PAC) were responsible for managing and ensuring the quality of all HIV programs in the province and supported the sites through regular audits and supervisory visits. However prior to 2011, clinical coaching occurred separately from audit and supervision, and routine performance measurement and structured QI activities were not occurring.

### Intervention

**Fig. 1:**
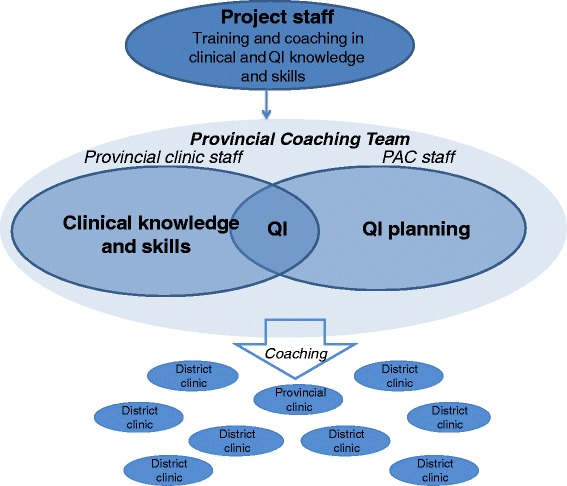
Model of Provincial-wide Capacity Building: Staff from the provincial clinic and staff from the Son La People’s AIDS Committee were trained in QI methodology and mentored to develop practical skills through implementation of QI at the Son La clinic by HAIVN staff. The provincial coaching team then provided coordinated clinical coaching and QI coaching to the district HIV clinics located throughout the province

Beginning in April 2011 our organization, The Partnership for Health Advancement in Vietnam (HAIVN), partnered with the Son La PAC to train members of the PCT to integrate QI coaching into their existing clinical mentoring, auditing, and supervision responsibilities. The PCT included a physician from the Provincial hospital clinic to provide HIV clinical and QI coaching to the district-level clinics, and staff members from the PAC to provide QI coaching, as well as to plan and implement the provincial-wide QI program (Fig. 1). Staff from the PAC and all staff working in the Son La Provincial clinic (including the physician identified to be the clinician member of the PCT) were trained in QI methodology through a two-day skills-building workshop. The workshop covered principles of quality and QI, indicator development, routine performance measurement, data collection and utilization, and QI project planning and implementation. Following the initial training, attendees gained hands-on experience supported by coaching from HAIVN staff to complete a baseline measurement of HIV quality in the provincial clinic.

### Study design

We conducted a prospective evaluation of the quality of care in seven HIV clinics before and after implementation of the coaching activities by the PCT.

### Performance measurement and population

To measure quality of care, core indicators were developed from existing international standards and national QI programs [25, 26]. Indicators included documentation of TB screening, adherence screening, whether eligible patients received cotrimoxazole (CTX) prophylaxis, CD4 cell count testing done in the past 6 months, routine lab monitoring (hemoglobin (Hgb), alanine aminotransferase (ALT)) in the previous 6 months, antiretroviral therapy (ART) start within 30 days of determination of clinical eligibility, and visit adherence. The visit adherence indicator changed after the first measurement at the clinic in Son La Provincial Hospital (“any missed visits in the previous year”) to “any missed visits in the previous 6 months” in all subsequent measurements done with HAIVN support through February 2013. Documentation of hepatitis C virus (HCV) screening was added as an indicator after the first measurement at the Son La Provincial Hospital clinic.

Data were extracted by chart review of a systematic sampling of patients who were active in care, defined as one or more visits in the previous 3 months for patients on ART or 6 months for patients not on ART. The sample size in each facility was calculated to be able to measure performance rates at the clinic level with a 95 % confidence interval +/−10 % for indicators with a 50 % rate. Data were extracted from charts every 6 months by clinic staff with coaching and oversight from PAC staff.

### Quality improvement

Following the initial quality measurement, HAIVN staff coached the PCT and provincial clinic staff to review and interpret the results, prioritize which gaps to improve, determine root causes, and develop and implement QI projects. This coaching was done through both on-site visits and by telephone. Coaching was initially provided every 1 to 2 months decreasing to quarterly after the first 6 months. Targeted clinical coaching was also provided by HAIVN staff to the Son La PCT and focused on improving clinical skills gaps identified in the performance measurement.

Once the PCT had gained skills in measurement, QI implementation, and clinical and QI coaching, HAIVN supported them to train district clinic staff in QI and provide follow-up clinical and QI coaching. In July 2011, the PCT began to train and coach the first three district clinics on performance measurement and QI. Three months later, three additional clinics were added. During quarterly provincial visits and regular phone conversations, HAIVN supported the members of the PCT by reviewing results, progress, and barriers in QI implementation and providing coaching on how to address identified challenges. This support was designed to further build the PCT’s capacity to strengthen QI efforts throughout the province (Fig. 1).

Three of the district clinics and the Provincial clinic completed a baseline, 6-month, and 12-month performance measurement. Three additional clinics had reached 6 months of follow up by the end of 2012. QI support in the remaining two district clinics was implemented in 2013, occurring just as the entire provincial QI program was selected to transition to the expanding national QI effort.

### Integration into Vietnam National QI Program

In early 2013, the VAAC began to roll out a national QI program for HIV care and treatment clinics. The HAIVN-supported provincial QI efforts transitioned over to the national QI program in February 2013. At this time, staff of the Son La provincial clinic and the six district clinics repeated a quality measurement using the national performance indicators. The only indicator definition that changed from the provincial measures already in use was visit adherence (from presenting as scheduled for all visits in the previous 6 months to only requiring presentation at the most recent scheduled visit). The national indicators also did not include routine Hgb, liver function tests or HCV testing.

After this performance measurement, staff from the clinics attended a national QI training. This training served as the initial introduction to QI for staff that joined the clinic after 2012 and refresher for previously trained staff. Following this training, the existing Son La PCT continued to support all clinics on a quarterly basis to review performance gaps, provide relevant QI and clinical skills coaching, and to develop and implement QI projects and plans. HAIVN staff remained available for telephone support to the PCT and accompanied the national technical working group on one visit to the province after the training.

### Analysis

We did a pre-post analysis of the change in performance measurement at the Provincial clinic and the first six district clinics before and after implementation of the PCT model and following transition to the national program. Performance rates were compared between the baseline, 6 and 12-month measurement using a chi-squared test. *P* value of < 0.05 was considered statistically significant. Visit adherence is only reported for the initial phase (pre-transition) because of the change to the national indicator. Data analysis was performed using STATA software version 12.

### Ethical considerations

The work was exempted from Institutional review as a quality improvement activity by both the U.S. Centers for Disease Control and Prevention and the Beth Israel Deaconess Medical Center IRB and was approved by the Vietnam Administration for AIDS Control. To ensure patient confidentiality, no patient identifiers were extracted during chart review and only unique study codes were entered into the database.

## Results

### Performance Measurement and QI at Son La Provincial Hospital Clinic

#### Baseline quality of care

**Fig. 2:**
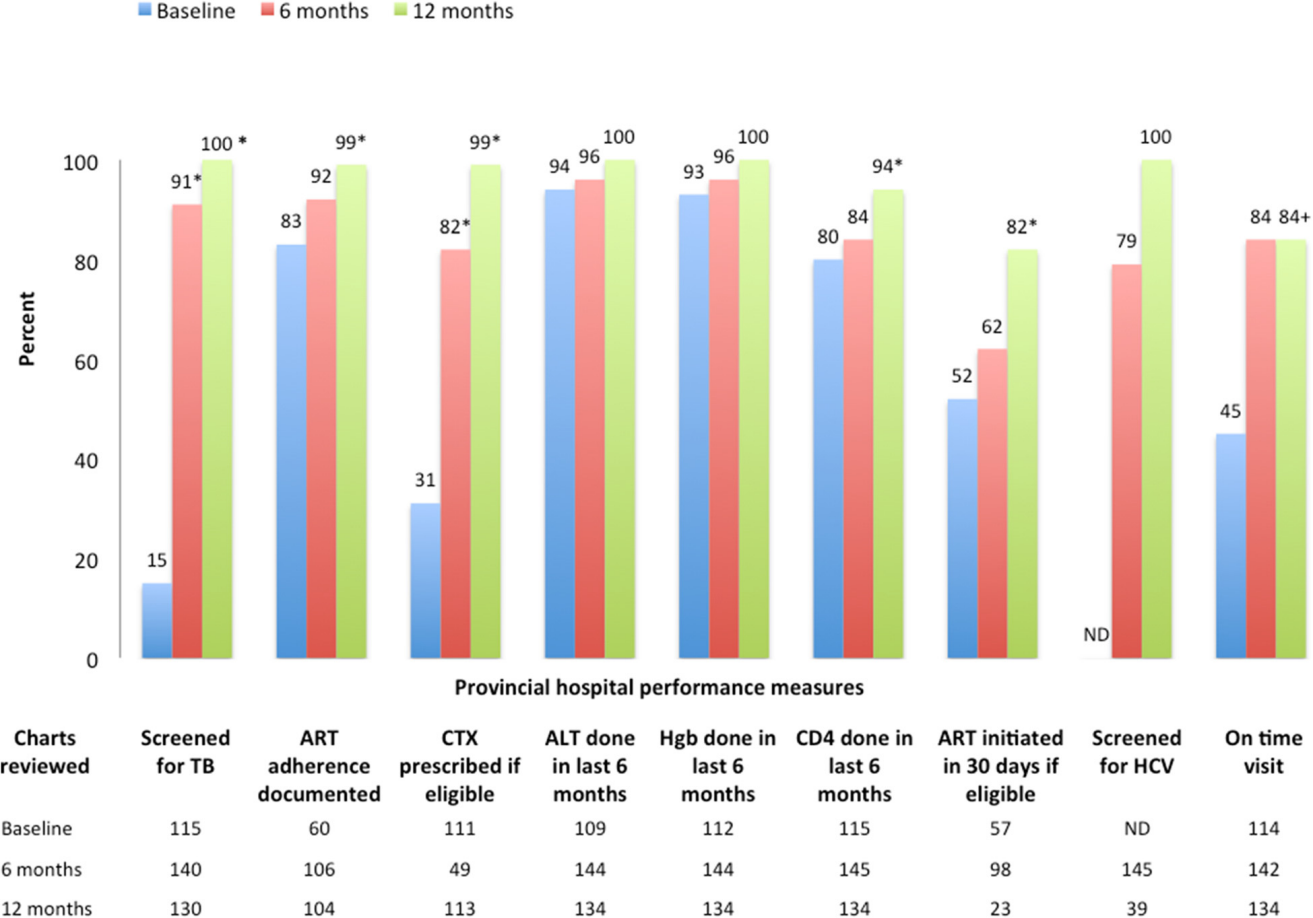
Quality of Care in Son La Provincial Clinic at baseline (after clinical training and coaching alone), 6 and 12 months after adding a quality improvement coaching program. (* indicates *p* < .05 compared to baseline, + indicates significance not calculated due to change in indicator definition). ND: Not done. ALT: Alanine aminotransferase

Despite a well-trained and experienced HIV clinic team, the baseline measurement identified several quality gaps at the provincial clinic (Fig. 2). The majority (83 %) of patients had documented medication adherence screening, over 90 % of patients had appropriate ALT and Hgb monitoring, and 80 % of patients had CD4 testing in the previous 6 months. However, only 15 % of patients were screened for symptoms of TB, 31 % of eligible patients received cotrimoxazole, and 52 % of patients in the previous 6 months started ART within 30 days of clinical eligibility. Fewer than half (45 %) of patients had come on time to their visits in the previous year.

#### QI

The Provincial clinic staff identified several possible root causes for the gaps, including poor chart documentation, and designed QI projects after the first measurement to improve three prioritized areas: TB screening, missed visits, and chart documentation. To improve TB screening, the clinic made use of a checklist of the four screening symptoms from the national guidelines (cough, fever, weight loss, night sweats) that was stamped into the chart by the nurse at the beginning of each clinic visit and served as a prompt for the examining physician. At the same time the data supporting the use of screening symptoms in active TB case finding were presented by HAIVN project staff in a brief lecture. These interventions led to rapid improvement in TB screening, which was maintained in future measurements. To decrease the number of missed visits, the clinic staff focused on improving their outreach to patients when they came late or missed their appointments. Through frequent checks of a small number of charts from patients seen in the previous week, the clinic staff was able to monitor whether their plans were having an impact and make adjustments where needed.

After the second performance measurement at 6 months, there was an upward trend in performance across all indicators. TB screening increased to 91 % of charts, cotrimoxazole prophylaxis was documented in 82 % of eligible patients, and CD4 testing was documented in 84 % of patients. By 12 months, TB screening, routine lab monitoring, and HCV screening reached 100 %, and statistically significant improvement was seen in all of the remaining indicators whose definitions remained constant (Fig. 2).

### Spread of QI to district clinics by the Provincial Coaching Team

**Fig. 3:**
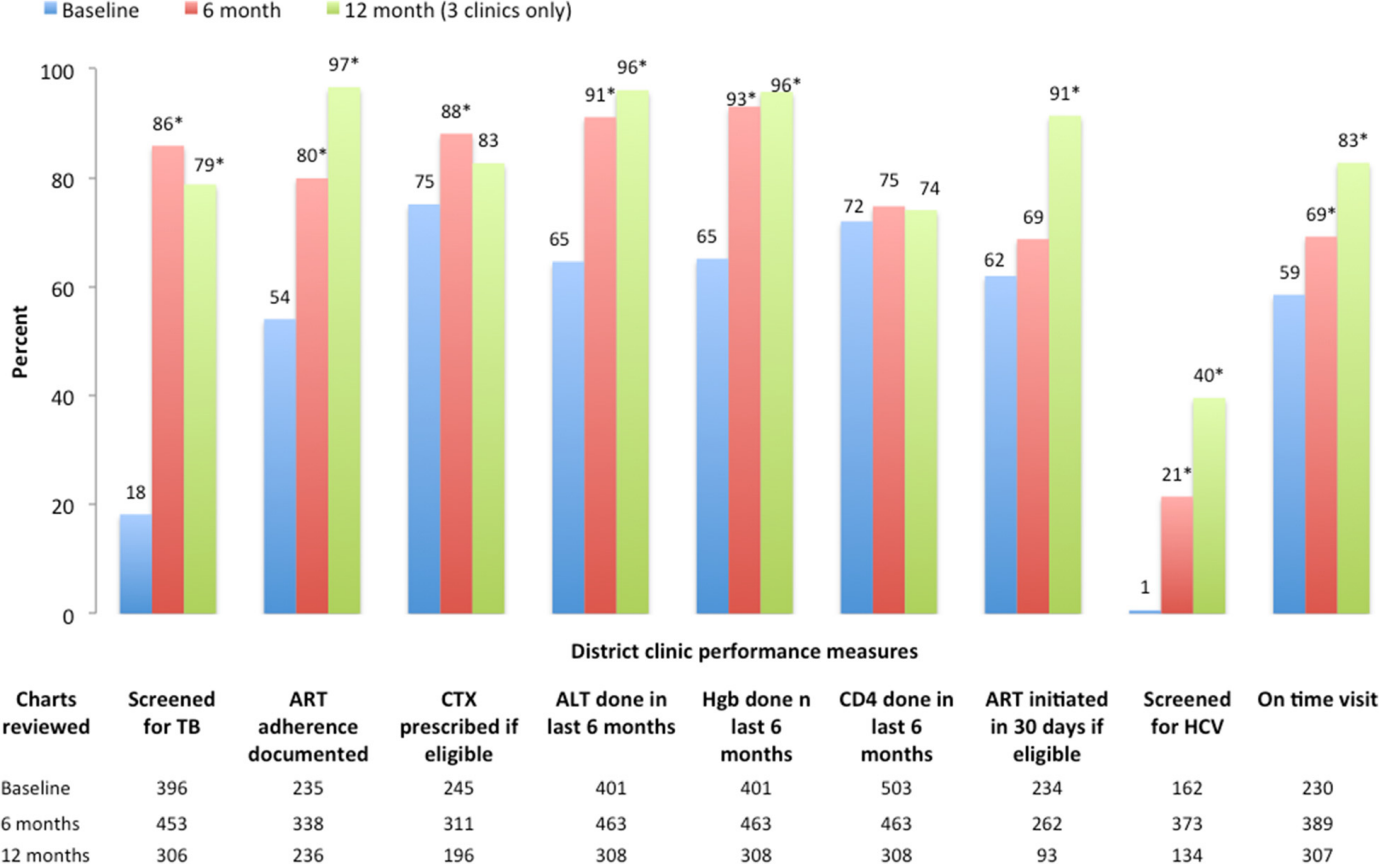
Quality of Care in Six District Outpatient Clinics coached by the Son La provincial coaching team at baseline, 6 and 12 months after implementation of a provincial QI program (* indicates *p* < .05 compared to baseline)

Across district clinics, the baseline performance measurement revealed gaps in TB screening (18 %), cotrimoxazole prophylaxis (75 %), and ART initiation within 30 days (62 %). Documentation of adherence screening was found in 54 %, and lab monitoring and CD4 testing in the last 6 months was found in 65 and 72 % of patients, respectively (Fig. 3). QI training and baseline measurements at the district clinics were followed by quarterly on-site visits to the clinics and periodic phone calls by the PCT to assist clinic staff as they implemented their interventions. Clinical coaching was provided by the Son La clinic HIV physician to the district clinic staff and tailored based on gaps in clinical practice identified from the measurement results. By December 2012, these six district clinics were actively implementing QI interventions and completed an average of five QI projects with the most frequent areas including improving lab testing, TB screening, and decreasing missed or late visits (Table 1).

**Table 1:** QI projects implemented by Son La HIV clinics

Priority gaps to improve	Description of QI projects implemented
Lab testing (ALT, hemoglobin and CD4) (6 clinics)	- Place template for monitoring routine testing in all patient charts
	- Review which patients will need lab testing prior to clinic
	- Schedule patients who will require similar testing on the same day
	- Review reasons for routine testing with patients
	- Contact patients who will need lab tests 1–2 days in advance
	- Make specific date for doing CD4 test each quarter
	- Review all patients who need to do CD4 test
TB screening (5 clinics)	- Use TB stamp or write 4 TB symptoms screening questions in chart
	- Remind staff in weekly meetings.
	- Place reminders on doctor’s desk to record TB screening
Missed visits (5 clinics)	- Logbook created and used to monitor missed visits
	- Follow up missed visits with phone call or home visit (treatment supporters or nursing staff)
	- Improve counseling on importance of attending scheduled clinic visits for patients starting and on ART
	- Update patients’ contact information at each visit
	- Group patients and schedule on the same day each month to more easily monitor for missed visits.
CTX prophylaxis (2 clinics)	- Review all eligible patients not yet on CTX
	- Update clinic staff on new guideline on CTX prophylaxis
	- Make plan for preparing CTX in advance
	- Document in patient charts if patient buys CTX at outside pharmacy
ART initiation within 30 days (2 clinics)	- Make a list of eligible patients to contact and remind them to come to clinic for ART treatment
	- Improve counseling prior to ART eligibility for patient at each visit
Patient chart documentation (4 clinics)	- Review required elements of chart documentation at staff meetings
	- Improve organization and filing of charts, and ensure all charts have complete patient identification number.
	- Review and complete demographics, treatment summary and test monitoring pages for all patient charts
	- Review patient charts at the end of each clinic day to finalize information

By the 6-month measurement, the overall quality in the six district clinics had significantly improved (*p* < .05) in TB screening, adherence counseling, cotrimoxazole prophylaxis, Hgb and ALT monitoring, HCV screening, and on-time visits (Fig. 3). The first three clinics also completed a 12-month measurement prior to roll out of the national program, with further improvement seen in nearly all of the indicators. ART initiation within 30 days reached 91 %, and on-time visits in the previous 6 months increased to 83 % from the average baseline of 65 % (in these three clinics). Not everything improved, with CD4 testing in the previous 6 months remaining stable at 74 % and HCV screening improving only to 40 % in the three clinics completing a 12-month measurement (Fig. 3).

### Sustained Quality and QI coaching Following Integration into the National QI Program

**Fig. 4:**
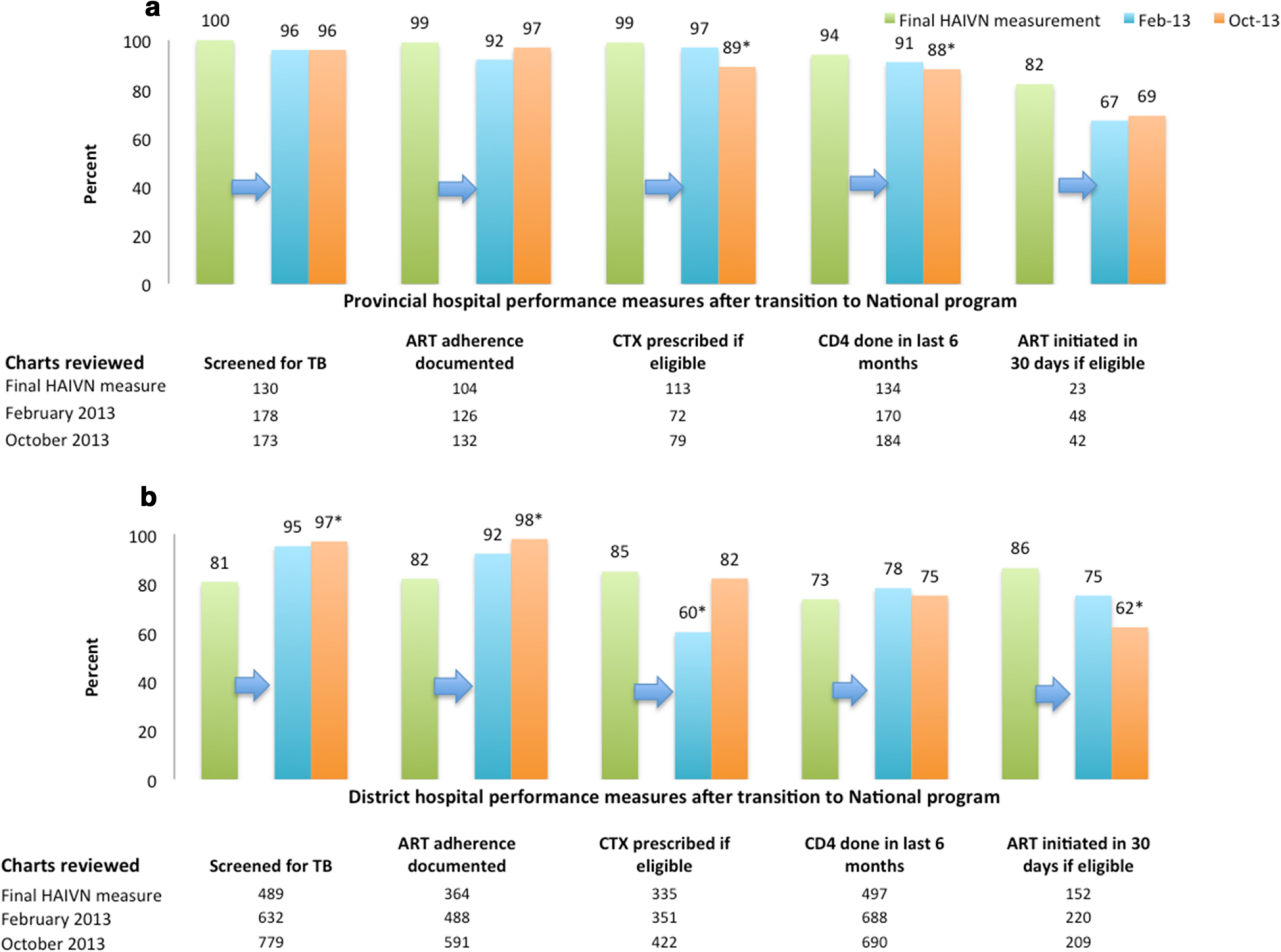
Change in Quality of Care from Final HAIVN-supported measurement through integration into the Vietnam National QI Program in February 2013. (**a**) Son La provincial hospital (final HAIVN measurement in June 2012) and (**b**) six district clinics (final HAIVN measurement between June and August, 2012)

Six months following the integration of the Son La QI efforts into the National Program, the PCT continued to independently support QI activities in the HIV clinics throughout the province. TB screening and adherence measurement remained high in the provincial clinic and improved in the district clinics (Fig. 4a and b). CD4 testing declined slightly in the final measurement at the provincial clinic (Fig. 4a). In the district clinics, routine CD4 testing (75 %) remained essentially unchanged in all measurements. Cotrimoxazole prophylaxis rates declined in both provincial and district clinics. While the rates were initially maintained in the provincial clinic, they dropped slightly at 12 months (97 to 89 %, *p* < .05). Cotrimoxazole prophylaxis dropped at six months (85 to 60 %, *p* < .05) in the district clinics (Fig. 4b). This decline was attributed to a supply chain gap and performance in the district clinics increased to 82 % in the most recent measurement. ART initiation within 30 days dropped in both the provincial and district clinics although the change at the provincial clinic did not reach statistical significance.

## Discussion

We describe the effectiveness of a model to build capacity of a team of existing public health sector staff to improve the quality of HIV care in a remote mountainous province in Vietnam. The model built critically important leadership and capacity for quality monitoring and QI coaching within in the provincial structure responsible for overseeing HIV care, resulting in a culture for change in the clinics. The approach of building on existing public-sector infrastructure and responsibilities ensured the development of a team able to effectively and rapidly lead the scale-up of QI. The model led to improved quality of HIV care across multiple remotely located district clinics without additional staffing. In addition, the approach of integrating into existing public sector infrastructure allowed the work to successfully transition to the national HIV QI program, with no interruption of the on-site support provided by the PCT, ongoing performance measurement, QI activities, and continued quality in a number of areas.

The model represented a change from the supervision and auditing previously performed by provincial level staff to active data-driven coaching that empowered clinic staff to identify and address gaps. The combined clinical and QI coaching helped improve identified knowledge and skills gaps while building capacity for needed systems and culture change. This approach of linking data feedback with action plans has been identified as critical to making supervision more effective [27]. The approach also allowed the PCT to prioritize efforts to sites with the lowest performance and to tailor activities based on identified gaps. This prioritization is critically important in the setting of limited resources and the travel time required for on-site visits.

Some identified gaps lent themselves to simple local solutions that resulted in rapid improvements. Poor documentation of care provided was a common gap across all of the district clinics, and an initial focus on appropriate documentation contributed to improvement in multiple indicators including adherence assessment and lab testing. The implementation of the TB stamp as a simple tool to improve screening was very successful. This tool was shared by the coaching team and quickly adopted by other HIV clinics, accelerating the improvement process and highlighting the advantage of a provincial-wide approach able to facilitate peer-to-peer learning.

However, gaps from larger systems issues were more challenging to improve and maintain at the clinic level. For example, CD4 testing was performed at only one provincial-level lab and only once per week. If reagents were in short supply, a sample was not sent to the lab, or if a patient did not return for testing on the appropriate day, there were delays in the test being done, resulting in no improvement in the CD4 indicator at the district clinics and a decline in the final provincial clinic measurement. Similar challenges in lab capacity were seen in the difference in improvement in HCV screening. At the provincial clinic, HCV screening improved rapidly but only reached 40 % at the district level. ART start time also remains a challenging indicator to improve in this province due to broader factors including patient distance from the clinics. As QI has become increasingly integrated into the clinic activities and led by clinic staff, the PCT and PAC can shift their focus to those quality issues that require a provincial-level approach.

There were a number of limitations to this work common to many studies of change associated with QI. It is largely based on programmatic data and documentation in the record. While the use of medical records data is a widely accepted approach to quality measurement, some portion of the reported improvements may have been due to changes in documentation rather than improvement in practice [28]. Second, this model has shown sustainability of the PCT and in documented improvement at one year after integration into a national program. However, longer term benefit and ongoing leadership and support by the provincial team needs to be demonstrated. Finally, as with any pre-post study, we could not rule out other factors resulting in improved clinic quality. However, no large external factors at the provincial or district levels that could have resulted in cross-indicator improvement were identified.

## Conclusions

In conclusion, we found that integrating coaching to improve systems into more traditional clinical skills-focused training was effective at increasing efforts to address gaps and improve the quality of clinical care. The model of building a team based on existing public sector staff and responsibilities was associated with strengthened leadership and rapid spread of QI, the capacity to recognize and address provincial-level system gaps, and the ability to ensure a seamless transition to an expanding national QI program. Further studies should examine whether the model and associated QI work can be sustained over time and adapted for effective replication in other setting and disease areas. Effective spread of the PCT model could be of tremendous value to other resource-limited countries working towards strengthening systems to support universal access to quality healthcare.

## Abbreviations

ALT: Alanine aminotransferase
ART: Antiretroviral therapy
CTX: Cotrimoxazole
HAIVN: Partnership for Health Advancement in Vietnam
HCV: Hepatitis C virus
HCW: Health care worker
Hgb: Hemoglobin
PAC: Peoples’ AIDS Committee
TB: Tuberculosis
QI: Quality improvement
VAAC: Vietnam Administration for AIDS Control
